# Cultivar-Specific Quality and In Vitro Antioxidant Profiles of Pear Pastes

**DOI:** 10.3390/foods15091515

**Published:** 2026-04-27

**Authors:** Jiajia Niu, Yanan Liu, Ke Zhang, Wei Cui, Yunfeng Lu, Yuanming Xie, Sipu Zhang

**Affiliations:** 1 Horticultural Research Institute, Henan Acadeny of Agriculnural Sciences, Zhengzhou 450002, China; 305jiajia@163.com (J.N.); zhangke58115@126.com (K.Z.); cuiwei131659@163.com (W.C.); 2 College of Horticulture, Nanjing Agricultural University, Nanjing 211800, China; 17275888906@163.com; 3 School of Life Science, Nanyang Normal University, Nanyang 473061, China; yunflu@163.com; 4 Henan Pear Industry Deep Processing Engineering Technology Research Center, Shangqiu 476700, China; zm18911870140@163.com; 5 Ningling Guoyuangong Food Co., Ltd., Shangqiu 476700, China

**Keywords:** pear paste, cultivar specificity, quality evaluation, antioxidant properties, Maillard reaction, multivariate analysis

## Abstract

Pear paste is a traditional Chinese product valued for its lung-moistening and antitussive effects. This study systematically evaluated the quality attributes and in vitro antioxidant profiles of pear pastes prepared in 2023 from 11 cultivars harvested in Henan Province, China. Multivariate analysis showed that genotype was the primary determinant of final product quality, with PC1 explaining 84.1% of the variance. Total phenolic content (TPC) ranged from 1052.8 to 1997.6 mg/kg, and total flavonoid content (TFC) from 478.1 to 1747.9 mg/kg across cultivars. Four cultivars displayed distinct advantages: ‘Akizuki’ (pronounced Maillard browning), ‘Wanxiu’ (highest free amino acids, 29.82 mg/g), ‘Hongzaosu’ (highest TPC, 1997.6 mg/kg; TFC, 1747.9 mg/kg; 2,2-diphenyl-1-picrylhydrazyl (DPPH) radical scavenging activity, 88.9 μmol TE/g), and ‘Huangguan’ (highest sensory score, 83.33; clarity, 87.65%). Antioxidant capacity was governed by a synergistic network of native phenolics, flavonoids, and Maillard reaction products, with the 2,2′-azino-bis(3-ethylbenzothiazoline-6-sulfonic acid) (ABTS) and ferric reducing antioxidant power (FRAP) assays recommended for consistent evaluation. These findings highlight the critical role of cultivar selection in tailoring the color, flavor, antioxidant activity, and overall quality of pear paste.

## 1. Introduction

Consumer interest in traditional foods with putative health benefits has risen in recent years. Chinese pear paste, a thick concentrate obtained through prolonged simmering of pear juice, has regained attention as both a home remedy for cough and sore throat and a potential functional ingredient [1]. Its long-standing reputation for lung-moistening and antitussive effects is now being reexamined through modern nutritional science.

Pear paste contains a complex array of bioactive compounds, including total phenolic content (TPC), total flavonoid content (TFC), arbutin, chlorogenic acid, quercetin, sugars (fructose, sorbitol), organic acids, triterpenoids, and pectin [2,3,4]. Recent studies have linked its consumption to modulation of cough, inflammation, and gut-related metabolites [1,5]. During thermal processing, Maillard reaction products (MRPs) form and influence color, flavor, and antioxidant activity [1,2,6,7]. Understanding how raw-material characteristics and processing conditions shape pear paste quality is therefore critical for both research and industry.

Numerous studies have investigated the chemical composition and bioactivity of pears and their processed products. Inter-cultivar differences in phenolic profiles are well documented [8,9,10], and commercial pear pastes vary considerably in antioxidant activity and overall quality [2,11]. Cough-suppressing compounds have been identified in pear paste [4], and their multidimensional effects from molecular mechanisms to clinical applications have been systematically analyzed [5]. Processing methods affect color, polyphenol content, and antioxidant properties [1]; vacuum concentration can enhance quality and antioxidant capacity [12,13]; and both enzymatic and non-enzymatic browning occur during crushing and boiling [14,15]. The kinetics of the Maillard reaction, its impact on antioxidant capacity, and the bioactivity of melanoidins have been reviewed [6,7,16]. Amadori compounds exhibit metal-chelating properties [17], and various MRPs possess free-radical-scavenging capabilities [18,19,20].

Despite these advances, most studies have focused on only a few pear cultivars or commercial pear paste products [2,8,9,10,11]. Systematic evaluations of locally grown cultivars in major production areas, particularly Henan Province, are lacking. The relationships between cultivar traits (sugars, acidity, free amino acids, phenolics), MRP formation during processing, and the resulting color, flavor, and antioxidant capacity remain unclear.

Henan Province is one of China’s three largest pear-producing regions, yet its processing industry lags behind the fresh fruit market. The three most widely cultivated cultivars are ‘Suli’, ‘Hwanggeum’, and ‘Akizuki’. Building on a multi-year evaluation of 21 cultivars that identified 10 candidates well suited to Henan’s climate [21], and considering maturation diversity (early, mid, late) alongside these major cultivars, we selected 11 cultivars for this study: ‘Zhongli No. 1’, ‘Huangguan’, ‘Wonhwang’, ‘Hwanggeum’, ‘Hwasan’, ‘Yuluxiang’, ‘Hongzaosu’, ‘Suli’, ‘Hongxiangsu’, ‘Wanxiu’, and ‘Akizuki’. Standardized pear pastes were prepared under controlled conditions and evaluated for appearance (color parameters), nutritional components (soluble sugars, titratable acidity, soluble protein, vitamin C), bioactive constituents (TPC, TFC), antioxidant capacity [2,2-diphenyl-1-picrylhydrazyl (DPPH), 2,2′-azino-bis(3-ethylbenzothiazoline-6-sulfonic acid) (ABTS), ferric reducing antioxidant power (FRAP)], free amino acids, and key MRPs (furfural, Strecker aldehydes). Multivariate analyses, including principal component analysis (PCA) and partial least squares discriminant analysis (PLS-DA), were employed to classify the samples and relate cultivar traits to antioxidant profiles.

This study offers the first comprehensive comparison of 11 pear cultivars specifically for pear paste production, integrating multiple quality dimensions (color, nutrition, bioactives, antioxidant capacity, free amino acids, and MRPs) within a single framework. The findings provide an evidence base for cultivar selection to enhance product quality, consistency, and market competitiveness, and clarify the synergistic interplay between native phenolics and flavonoids and processing-derived MRPs in regulating antioxidant capacity.

## 2. Materials and Methods

### 2.1. Chemicals and Reagents

All chemicals and reagents used were of analytical grade and purchased from commercial suppliers in China, including Shanghai Aladdin Biochemical Technology Co., Ltd., Tianjin Kemiou Chemical Reagent Co., Ltd. (Tianjin, China), and Shanghai McLean Biochemical Technology Co., Ltd. (Shanghai, China). Sodium chloride (chromatography grade) and 2-methyl-3-heptanone (chromatography grade) were obtained from Shanghai Maclin Biochemical Technology Co., Ltd. (Shanghai, China), and Merck KGaA (Darmstadt, Germany), respectively.

### 2.2. Plant Preparation and Treatment

Fruits from eleven pear cultivars were harvested at commercial maturity in 2023 (Table 1). ‘Suli’ pears were harvested from Shiqiao Town, Ningling County, Shangqiu City, Henan Province (34°30′12″ N, 115°18′07″ E). All other cultivars were obtained from the Pear Demonstration Garden at the Henan Modern Agricultural Research and Development Base (35°00′19″ N, 113°42′16″ E). For each cultivar, fruits uniform in color, size, and maturity, and free from defects, were selected.

For each cultivar, three representative trees were selected as biological replicates; these trees had similar trunk circumferences, crown volumes and fruit yields, and were free from obvious diseases. On the day of commercial harvesting, fruits of uniform colour, size and ripeness, and free from blemishes, were selected for each variety. Approximately 5 kg of fruit was harvested from each tree within each replicate and stored in cold storage. Once harvesting was complete for all cultivars, 10 fruits were randomly selected from each replicate for subsequent experiments.

The fruits were washed to remove surface debris. A standardized processing method was applied: fruits were crushed and juiced using a Z12-LZ566 juicer (Jiuyang Original, Hangzhou Jiuyang Small Appliances Co., Ltd., Hangzhou, China). The juice was filtered through a 200-mesh sieve. The filtrate was heated to boiling on a 1200 W induction cooker, then the power was reduced to 300–600 W to maintain a temperature of approximately 100–120 °C. The juice was concentrated with constant stirring until the soluble solids content (TSS) reached 60%. Final concentration was achieved under vacuum (0.096 MPa) at 70 °C using a rotary distillation flask (100 rpm) until a TSS of 77–78% was obtained.

Once the pear paste had been prepared, all samples were stored in sealed containers at 25 °C and analysed within two months. For each cultivar, three biological replicates were prepared, each derived from a single tree. From each biological replicate, three technical replicates were measured for each assay.

### 2.3. Determination of Quality Indices for Pear Paste

#### 2.3.1. Titratable Acidity (TA)

TA was determined by means of automatic potentiometric titration. The pear paste was diluted 10-fold with distilled water. A 1 mL aliquot of the diluted sample was mixed with 24 mL of distilled water, and one drop of 1% (*w*/*v*) phenolphthalein solution was added as an indicator. The mixture was titrated with 0.05 mol/L NaOH using a ZDJ-4B automatic titrator (Ray Magnetic, Shanghai, China). The titratable acidity was calculated using the conversion factor for malic acid (0.067) and expressed as a percentage of malic acid equivalents.

#### 2.3.2. Soluble Sugars (SS)

SS content was determined using the anthrone-sulfuric acid method [11] with minor modifications. Pear paste was diluted 1000-fold with distilled water and centrifuged at 10,000× *g* at 4 °C for 20 min. A 1.0 mL aliquot of supernatant was mixed with 2.0 mL of anthrone-sulfuric acid reagent, heated in a boiling water bath for 10 min, cooled, and its absorbance measured at 620 nm. A glucose standard curve (10–100 µg/mL) was used for quantification.

#### 2.3.3. Soluble Protein (SP)

SP content was determined using the Coomassie Brilliant Blue G-250 method [22]. A standard curve was prepared with bovine serum albumin (BSA, 0.25–2.5 µg/mL). Pear paste was diluted 10-fold, ultrasonically extracted at 40 °C for 20 min, and centrifuged at 10,000× *g* for 20 min. The supernatant was mixed with the reagent, and absorbance was measured at 595 nm.

#### 2.3.4. Determination of Pear Fruit Quality Indicators

Flesh firmness was measured at predefined positions on peeled fruit with a penetrometer (GS-15, GUSS, Strand, South Africa) fitted with an 11 mm probe. After juice extraction, the juice was filtered through four layers of gauze. The total soluble solids (TSS) of the filtered juice were immediately determined using a PAL-1 digital refractometer (Atago, Tokyo, Japan). The titratable acidity (TA) of the juice was determined according to the method outlined in Section 2.3.1. The juice yield was calculated as follows: Juice yield (%) = (Weight of extracted juice/Total weight of fruit material used for juicing) × 100.

### 2.4. Determination of Color and Appearance of Pear Paste

Following a reported method [23], pear paste was diluted with distilled water to 11.5% TSS for analysis.

#### 2.4.1. Color Parameters

Color parameters were measured using a CR-400 colorimeter (Konica Minolta, Tokyo, Japan) equipped with illuminant D65 and a 10° observer angle. The instrument was calibrated against a white standard plate before the start of each measurement session. For each pear paste sample, three independent replicates were prepared. From each replicate, a 20 mL aliquot of the diluted pear paste was placed in a colorless, transparent Petri dish, which was then positioned inside a light-tight, opaque box to exclude external light interference. Three successive readings were taken per replicate, and the average values were calculated. The colorimeter directly recorded the values of L^*^ (lightness: 0 = black, 100 = white), a^*^ (green-red axis), b^*^ (blue-yellow axis), C^*^ (chroma), and *h*° (hue angle).

#### 2.4.2. Clarity

The solution was centrifuged at 10,000× *g* for 10 min. The transmittance of the supernatant at 625 nm was measured using a spectrophotometer, with distilled water as the blank [24].

### 2.5. Determination of the Antioxidant Indices of Pear Paste

#### 2.5.1. Total Phenolic Content (TPC)

The total phenolic content (TPC) was determined using the Folin–Ciocalteu method with modifications based on Albarri et al. [25]. Briefly, the pear paste was initially diluted 10-fold with distilled water. Subsequently, a 0.5 mL aliquot of the diluted sample was mixed with 2.5 mL of distilled water and 2.5 mL of Folin–Ciocalteu reagent. The mixture was vortexed and allowed to stand in the dark for 1 min. Then, 2 mL of a 20% (*w*/*v*) sodium carbonate (Na_2_CO_3_) solution was added. After thorough mixing, the reaction mixture was incubated in a water bath at 30 °C for 60 min. The absorbance was measured at 760 nm using a TU-1950 UV-Vis spectrophotometer (Beijing, China). Gallic acid solutions with concentrations ranging from 10 to 50 μg/mL were used to establish a standard curve for quantification.

#### 2.5.2. Total Flavonoid Content (TFC)

The total flavonoid content (TFC) was determined according to the method of Albarri et al. [25] with slight modifications. Briefly, the pear paste was diluted 10-fold with distilled water. A 0.5 mL aliquot of the dilution was sequentially mixed with 3.5 mL of 60% (*v*/*v*) ethanol, 0.3 mL of 5% (*w*/*v*) sodium nitrite, and 0.3 mL of 5% (*w*/*v*) aluminum nitrate, with a 6 min incubation after the addition of each reagent. Subsequently, 4 mL of 4% (*w*/*v*) sodium hydroxide (NaOH) was added. The reaction mixture was vortexed, incubated at room temperature for 15 min in the dark, and the absorbance was then measured at 510 nm using a TU-1950 UV-Vis spectrophotometer (Beijing, China). A standard curve was constructed using rutin solutions within a concentration range of 5–500 μg/mL for quantification.

#### 2.5.3. Vitamin C Content

The vitamin C (Vc) content was determined using the molybdenum blue colorimetric method according to Niu et al. [26] with modifications. Briefly, the pear paste was diluted 10-fold with an oxalic acid-EDTA solution and centrifuged at 10,000× *g* for 10 min. A 1.0 mL aliquot of the supernatant was then mixed sequentially with 0.1 mL of 3% (*w*/*v*) metaphosphoric acid–acetic acid solution and 0.2 mL of 5% (*v*/*v*) sulfuric acid. After vortexing, 0.4 mL of 5% (*w*/*v*) ammonium molybdate solution was added. The reaction mixture was incubated at room temperature for 15 min, and the absorbance was measured at 705 nm using a TU-1950 UV-Vis spectrophotometer (Beijing, China). A standard curve was constructed using L-ascorbic acid solutions with concentrations ranging from 20 to 500 μg/mL for quantification.

#### 2.5.4. Sensory Evaluation

Sensory evaluation was conducted by a trained panel consisting of nine members (four males and five females), aged between 25 and 50 years, all of whom had prior experience in sensory evaluation of fruit-based products. A standardized scoring system was employed, evaluating four key attributes: taste (30 points), aroma (30 points), color (20 points), and texture (20 points), with a maximum total score of 100. The weight distribution was designed based on the relative importance of each attribute to the overall quality perception of pear paste, with taste and aroma considered the primary contributors. The detailed scoring criteria for each attribute are summarized in Table 2. Briefly, a higher score indicates better performance in terms of flavor balance, aroma purity, color appeal, and texture consistency. All samples were presented in random order under controlled lighting conditions, and panelists were instructed to rinse their palates with water between evaluations [12].

Before starting the evaluation, the research team explained the scope and details of the project to the participants, including the purpose of the research, the identity of the researchers, data protection measures, privacy and data retention policies, the voluntary nature of participation, the right to withdraw at any time, and contact details for any questions that might arise. Finally, all participants signed a written informed consent form, confirming that they had read and understood the information provided, and that their questions had been answered.

### 2.6. Determination of Antioxidant Properties of Pear Paste

#### 2.6.1. DPPH Radical Scavenging Rate (DPPH RSR)

The DPPH radical scavenging activity was measured following a modified procedure based on Li et al. [12]. Briefly, the pear paste was diluted 10-fold and centrifuged at 10,000× *g* for 10 min. Then, 0.2 mL of the supernatant was mixed with 3.8 mL of a 0.1 mM ethanolic DPPH working solution. The control was prepared by mixing 0.2 mL of anhydrous ethanol with 3.8 mL of the DPPH solution, and the background by mixing 0.2 mL of the supernatant with 3.8 mL of anhydrous ethanol. All mixtures were vortexed, incubated in the dark at room temperature for 45 min, and their absorbance was measured at 517 nm using a TU-1950 UV-Vis spectrophotometer. Antioxidant capacity was quantified using a Trolox standard curve (100–800 μmol/L) and expressed as μmol Trolox equivalents per gram (μmol TE/g).

#### 2.6.2. ABTS Radical Scavenging Rate (ABTS)

The ABTS radical scavenging activity was measured based on a modified method of Zhang et al. [13]. Briefly, the pear paste was diluted 10-fold and centrifuged at 10,000× *g* for 10 min. Then, 0.2 mL of the supernatant was mixed with 3.8 mL of ABTS working solution. The control contained 0.2 mL of distilled water mixed with 3.8 mL of ABTS solution, and the background contained 0.2 mL of the supernatant mixed with 3.8 mL of distilled water. After vortexing, all mixtures were incubated at room temperature in the dark for 20 min, and absorbance was measured at 734 nm. Antioxidant capacity was quantified using a Trolox standard curve (200–1400 μmol/L) and expressed as μmol Trolox equivalents per gram (μmol TE/g).

#### 2.6.3. Ferric Reducing Antioxidant Power (FRAP)

The ferric reducing antioxidant power (FRAP) was measured following the method described by Li et al. [12]. Briefly, the pear paste was diluted 10-fold and centrifuged at 10,000× *g* for 10 min. A 1.0 mL aliquot of the supernatant was mixed with 1.0 mL of phosphate buffer (0.2 M, pH 6.6) and incubated in a water bath at 50 °C for 20 min. The reaction was terminated by adding 2.5 mL of 10% (*w*/*v*) trichloroacetic acid. Subsequently, 2.5 mL of the mixture was transferred to a new tube, mixed with 0.5 mL of 0.1% (*w*/*v*) ferric chloride solution, and kept at room temperature for 10 min. The absorbance was measured at 593 nm using a TU-1950 UV-Vis spectrophotometer. Antioxidant capacity was quantified against a Trolox standard curve (100–800 μmol/L) and expressed as μmol Trolox equivalents per gram (μmol TE/g).

### 2.7. Determination of Maillard Reaction Products

#### 2.7.1. Browning Degree

Melanoidins, which are brownish polymers, are commonly expressed as the browning degree (BD). The sample treatment method was consistent with Section 2.3.2, and the absorbance was measured at a wavelength of 420 nm for quantification.

#### 2.7.2. Volatile Compounds Such as Aldehydes, Furans, and Their Derivatives

For products related to the Maillard reaction, such as aldehydes, furans, and their derivatives, a method slightly modified from Zhang et al. [13] was employed. A 1.0 g sample of pear paste was placed in a test tube, to which 0.20 g NaCl and 5 μL internal standard (2-methyl-3-heptanone, concentration 0.031 g/L) were added. Equilibrate the mixture at 60 °C under magnetic stirring for 20 min. Following equilibration, perform headspace adsorption with an SPME probe for 30 min, followed by chromatographic and mass spectrometric analysis. Chromatography conditions: HP-5MS capillary column (30 m × 0.25 mm, 0.25 μm); inlet temperature 260 °C; carrier gas He (purity 99.999%), flow rate 1 mL/min; splitless injection. Temperature program: Initial temperature 40 °C, held for 3 min, then ramped at 2 °C/min to 70 °C, followed by a 5 °C/min ramp to 150 °C, and finally an 8 °C/min ramp to 230 °C. Mass Spectrometry Conditions: Electron Impact Ionization (EI), electron energy 70 eV, ion source temperature 230 °C, interface temperature 230 °C, mass scan range *m*/*z* 35–500, solvent removal time 2 min. Qualitative analysis was performed by searching the acquired spectra against the NIST spectral library of the mass spectrometer. Matches exceeding 80% confidence were selected as the basis for substance identification. The absolute content of volatile substances was calculated based on peak area.

### 2.8. Determination of Free Amino Acids

Free amino acid content was determined according to the method of Huang et al. [27] using an amino acid analyzer (Hitachi L-8900, Tokyo, Japan). Briefly, 2.0 g of pear paste was homogenized with 20 mL of 3% (*w*/*v*) sulfosalicylic acid solution. The mixture was then centrifuged at 10,000× *g* and 4 °C for 15 min. The resulting supernatant was carefully collected, passed through a 0.22 μm microporous membrane filter, and then injected into the amino acid analyzer for quantification.

### 2.9. Data and Statistical Analysis

All measurements were performed in triplicate as technical replicates for each biological replicate. Three biological replicates were prepared per cultivar. The results are expressed as mean ± standard error of the mean (SEM) of the three biological replicates. Differences among means were evaluated by means of one-way analysis of variance (ANOVA) followed by Tukey’s HSD post hoc test, with a significance level set at *p* < 0.05. Relationships between continuous variables were assessed using Pearson’s correlation analysis. To discern multivariate patterns and group separation, Partial Least Squares Discriminant Analysis (PLS-DA) was applied to standardized data. Statistical analyses were performed using SPSS 20.0 (IBM, Chicago, IL, USA), while PLS-DA was carried out with SIMCA-P (version 14.1, Umetrics, Sweden). Figures were prepared using Origin 2024 (OriginLab Inc., Northampton, MA, USA).

## 3. Results

### 3.1. Visual Appearance of Different Cultivars of Pear Paste

**Figure 1:**
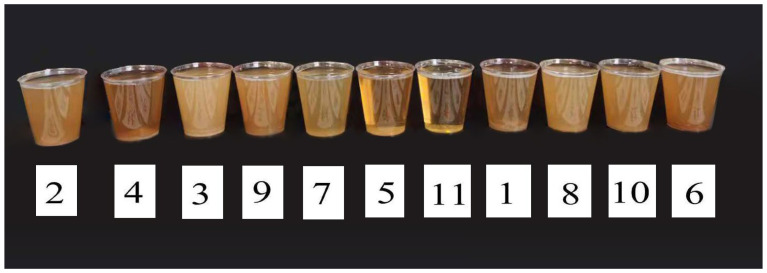
Visual appearance of reconstituted pear paste from different cultivars. Note: The numbers correspond to the cultivar serial numbers listed in Table 3.

Color parameters of pear paste varied significantly among cultivars (Table 3, *p* < 0.05). Lightness (L^*^) showed a broad range, with the highest values observed in ‘Hwasan’ and ‘Huangguan’ (*p* < 0.05 vs. other cultivars), while ‘Akizuki’ exhibited the lowest L^*^ (*p* < 0.05). The red–green axis (a^*^) revealed that ‘Akizuki’ and ‘Hongzaosu’ possessed the most intense red tones (*p* < 0.05), whereas ‘Huangguan’ and ‘Suli’ displayed the strongest greenish components (*p* < 0.05). For the yellow–blue axis (b^*^) and chroma (C^*^), ‘Wanxiu’ had the highest values (*p* < 0.05), indicating a vivid yellow color, while ‘Huangguan’ showed the lowest C^*^ and the highest hue angle (*h*°) (*p* < 0.05), confirming its yellowish appearance. Conversely, ‘Hongzaosu’ and ‘Akizuki’ had lower *h*° values (*p* < 0.05), corresponding to redder hues. Clarity (transmittance at 625 nm) varied substantially, with ‘Huangguan’ achieving the highest clarity (87.65%) (*p* < 0.05) and ‘Hwasan’ the lowest (3.33%) (*p* < 0.05). Overall, cultivar selection profoundly influenced the color and clarity of pear paste: ‘Huangguan’ was characterized by high lightness and clarity with a yellowish tone, whereas ‘Akizuki’ and ‘Hongzaosu’ were distinguished by redder hues (Figure 1).

### 3.2. Comparison of Nutritional Quality Indicators Across Different Cultivars of Pear Paste

Nutritional composition and sensory quality varied significantly among cultivars (Table 4, *p* < 0.05). Soluble sugars, TA, Vc, and soluble protein each exhibited wide inter-cultivar ranges, with distinct cultivars showing the extreme values for each parameter. Soluble sugars were significantly higher in ‘Akizuki’ than in all other cultivars (*p* < 0.05), whereas ‘Yuluxiang’ showed the lowest content (*p* < 0.05). TA peaked in ‘Hongzaosu’, which differed significantly from the rest (*p* < 0.05), while ‘Hongxiangsu’ and ‘Suli’ exhibited the lowest TA values with a significant difference between them (*p* < 0.05). Vc content was significantly higher in ‘Hwanggeum’ than in other cultivars (*p* < 0.05) and significantly lower in ‘Huangguan’ (*p* < 0.05). Soluble protein was greatest in ‘Suli’, significantly outperforming all others (*p* < 0.05), and lowest in ‘Huangguan’ (*p* < 0.05). TFC and TPC followed a similar trend, both being highest in ‘Hongzaosu’ (*p* < 0.05) and lowest in ‘Hwasan’ (*p* < 0.05). Sensory scores also differed markedly: ‘Huangguan’ received the highest score (*p* < 0.05), while ‘Hwasan’ received the lowest (*p* < 0.05), with several mid-scoring cultivars showing no significant differences among them (*p* > 0.05). Overall, cultivar selection profoundly influenced the nutritional and sensory properties of pear paste, with ‘Hongzaosu’ standing out for its high bioactive content and ‘Huangguan’ for its superior sensory quality.

### 3.3. Comparative Analysis of Maillard Reaction Products Among Different Cultivars of Pear Paste

Many volatile Maillard reaction products (MRPs) were not detected in several cultivars (indicated by “-” in Table 5). Due to large differences in concentration magnitudes, statistical comparisons were not performed for individual volatile compounds. Different cultivars exhibited marked variations in volatile MRPs. In contrast, the browning degree (BD) differed significantly among cultivars (Table 5, *p* < 0.05). Furfural was the predominant volatile in all samples. Total MRPs (M-TOTAL) varied widely, with the highest levels observed in ‘Yuluxiang’ and the lowest in ‘Wanxiu’. Strecker aldehydes (e.g., 2-methylpropanal, 3-methylbutanal, 2-methylbutanal, and methional) were detected only in selected cultivars, with ‘Akizuki’ and ‘Hongzaosu’ showing the greatest diversity and highest concentrations. Other MRP-derived volatiles, including 5-methylfurfural, benzeneacetaldehyde, 2-furanmethanol, pyrazines, and furanones, exhibited cultivar-dependent occurrence patterns. BD was highest in ‘Akizuki’ (*p* < 0.05) and lowest in ‘Huangguan’ (*p* < 0.05), with ‘Hongzaosu’ showing an intermediate value that differed significantly from both extremes (*p* < 0.05). The remaining cultivars formed three statistically distinct BD groups (*p* < 0.05). Overall, the Maillard reaction intensity and volatile profiles were strongly cultivar-dependent, with ‘Yuluxiang’ producing the highest total MRPs, ‘Akizuki’ the most intense browning, and ‘Huangguan’ the least browning.

### 3.4. Comparison of Amino Acid Content Across Different Cultivars of Pear Paste

Total amino acid (TAA), essential amino acid (EAA), and non-essential amino acid (NEAA) contents varied significantly among cultivars (Table 6, *p* < 0.05). TAA content showed a wide inter-cultivar range, with the highest values observed in ‘Wanxiu’ and ‘Akizuki’ (significant difference between them, *p* < 0.05) and the lowest in ‘Yuluxiang’, ‘Huangguan’, and ‘Hwanggeum’ (which did not differ significantly from one another *p* > 0.05). EAA content followed a similar pattern, with ‘Akizuki’ and ‘Wanxiu’ again exhibiting the highest values (*p* < 0.05 vs. others), indicating superior nutritional quality. Regarding amino acid diversity, ‘Akizuki’ contained the broadest spectrum (16 types), whereas ‘Suli’ had the fewest (11 types). ‘Yuluxiang’ showed the greatest diversity of essential amino acids (seven types). NEAA content followed the same trend as TAA, being significantly higher in ‘Wanxiu’ and ‘Akizuki’ than in other cultivars (*p* < 0.05). Overall, cultivar selection profoundly influenced both the quantity and diversity of free amino acids in pear paste, with ‘Wanxiu’ and ‘Akizuki’ distinguished by their high total and essential amino acid contents.

### 3.5. Comparison of Antioxidant Properties Across Different Cultivars of Pear Paste

**Figure 2:**
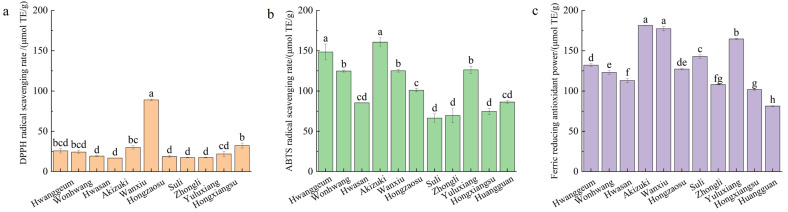
Comparison of antioxidant properties among different pear paste cultivars. (**a**) DPPH radical scavenging activity; (**b**) ABTS radical scavenging activity; (**c**) Ferric reducing antioxidant power (FRAP). All values are expressed as mean ± standard error (*n* = 3). Different lowercase letters above the bars indicate significant differences among cultivars at *p* < 0.05, as determined by means of one-way ANOVA followed by Tukey’s HSD post hoc test.

Antioxidant capacity, assessed using DPPH, ABTS, and FRAP assays, varied significantly among cultivars (Figure 2, *p* < 0.05). The three assays revealed distinct yet partially consistent response patterns, with FRAP showing the greatest inter-cultivar variation and DPPH the least. Notably, while DPPH values remained relatively low and similar across most cultivars (*p* > 0.05 among low-activity cultivars), ABTS and FRAP values exhibited a wider range and showed a strong positive correlation in cultivar ranking. In the ABTS assay, ‘Akizuki’ and ‘Hwanggeum’ exhibited the highest activity, with no significant difference between them (*p* > 0.05). The FRAP assay again ranked ‘Akizuki’ and ‘Wanxiu’ highest (*p* < 0.05 vs. others), while ‘Huangguan’ was ranked lowest (*p* < 0.05). Across all assays, FRAP consistently yielded the highest antioxidant values, followed by ABTS, whereas DPPH activity was generally the lowest, reflecting inherent differences in assay mechanisms. Clear clustering of high- and low-activity cultivars was evident: both FRAP and ABTS identified a group of cultivars with significantly higher activity (*p* < 0.05), whereas DPPH showed fewer statistically significant groupings. Overall, these three assays collectively confirmed that the antioxidant capacity of pear paste is strongly cultivar-specific. ‘Akizuki’, and ‘Wanxiu’ demonstrated excellent antioxidant activity across multiple assays (*p* < 0.05), while ‘Hwasan’, ‘Hongxiangsu’, and ‘Zhongli 1’ consistently ranked among the least active cultivars (*p* < 0.05). Among the assays, FRAP and ABTS were more sensitive and consistent than DPPH in distinguishing between cultivars.

### 3.6. Correlation Analysis of Indicators Across Different Pear Paste Cultivars

**Figure 3:**
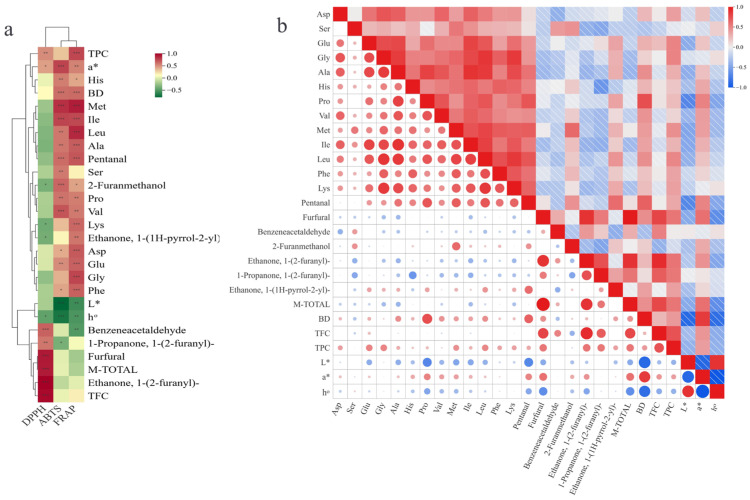
Correlation Cluster Analysis of Antioxidant Evaluation Indicators (**a**), Correlation Analysis Among Indicators (**b**). Note: Asterisks within the correlation matrix denote the significance level of correlations (* *p* < 0.05, ** *p* < 0.01, *** *p* < 0.001).

Correlation cluster analysis (Pearson, *p* < 0.05, |r| > 0.50) was performed on antioxidant activities (DPPH, ABTS, FRAP), color parameters (L^*^, a^*^, *h*°), free amino acids, antioxidant composition (TFC, TPC), Maillard-derived volatiles, and M-TOTAL (Figure 3a). The heatmap revealed distinct groupings: ABTS and FRAP clustered together, whereas DPPH formed a separate branch; L^*^ and a^*^ segregated with opposite gradients, consistent with their negative correlation. M-TOTAL grouped with Maillard volatiles (e.g., furfural, 2-furanmethanol) and several free amino acids. TFC and TPC clustered with antioxidant indices, and proline aligned with Maillard-related volatiles and browning indicators. The Pearson matrix (Figure 3b) visualized significant associations, with red dots denoting positive and blue dots negative correlations; dot size reflected correlation strength. L^*^ was strongly negatively correlated with a^*^ and BD. TFC correlated positively with DPPH, and TPC correlated positively with FRAP and multiple amino acids (e.g., leucine, lysine). ABTS was positively associated with amino acids (e.g., methionine, valine) and negatively with L^*^. M-TOTAL showed strong positive correlations with furfural and benzeneacetaldehyde; several amino acids (e.g., methionine, isoleucine) correlated positively with furanic and carbonyl flavor compounds. Histidine correlated negatively with pentanal, and most amino acids were positively intercorrelated, forming a cohesive positive network.

### 3.7. Comprehensive Evaluation and Ranking of Antioxidant Properties in Different Pear Paste Cultivars

**Figure 4:**
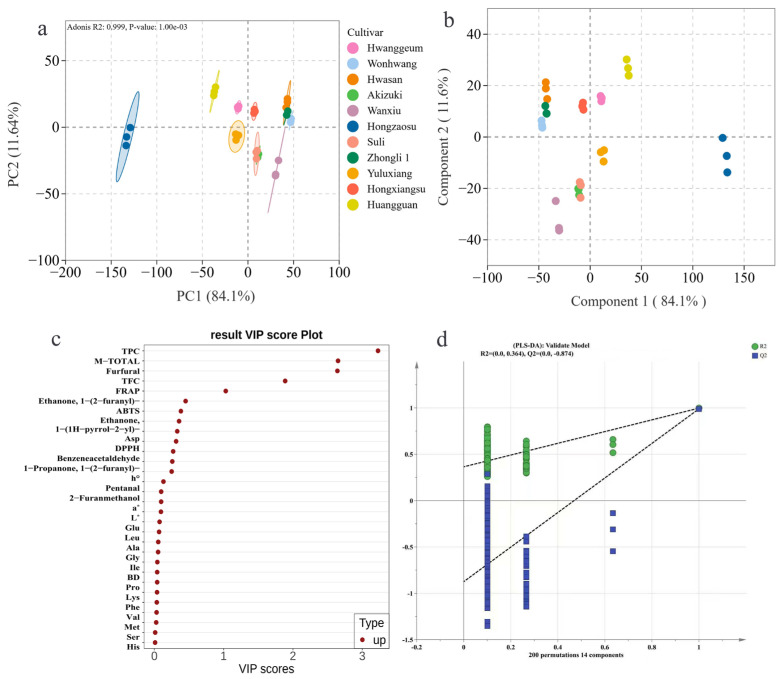
Multivariate statistical analysis of pear paste from different cultivars based on antioxidant and physicochemical indicators. PCA score plot (**a**), PLS-DA plot (**b**), VIP score plot screening key discriminatory variables (**c**), and PLS-DA Permutation Test (**d**).

Multivariate analyses were performed to discriminate pear paste samples from the 11 cultivars. A partial least squares discriminant analysis (PLS-DA) model was constructed and validated using 200-fold cross-validation (Figure 4d). The cross-validation plot yielded R^2^Y intercepts of 0.0 and 0.364, and a Q^2^ intercept of −0.874. Variable importance in projection (VIP) scores identified TPC (VIP > 3.0), M-TOTAL, furfural, and TFC as the primary markers distinguishing antioxidant and quality attributes (Figure 4c). The PLS-DA score plot revealed clear separation of cultivars, with principal component 1 (PC1) explaining 84.1% of the variance and PC2 explaining 11.6% (cumulative 95.7% Figure 4b). Distinct clustering patterns were observed: ‘Hongzaosu’ was located on the positive side of PC1; ‘Huangguan’ in the upper-right quadrant; ‘Wonhwang’ and ‘Zhongli 1’ on the left; ‘Wanxiu’, ‘Akizuki’, and ‘Suli’ in the lower-left region; and ‘Hwasan’ and ‘Yuluxiang’ in the upper-central and central areas, respectively. Minimal overlap was observed among biological replicates. Principal component analysis (PCA) confirmed these distribution patterns (PC1: 84.1%, PC2: 11.64%, cumulative 95.74%, Figure 4a). An Adonis test confirmed highly significant differences among cultivars (R^2^ = 0.999, *p* = 0.001). Similar clustering was observed: ‘Hongzaosu’ remained independent on the PC1 axis, ‘Wonhwang’ formed an independent cluster on the left, and the distribution of the remaining cultivars in the PCA space was consistent with the PLS-DA results.

## 4. Discussion

### 4.1. Cultivar Specificity as the Fundamental Driver of Pear Paste Quality

Multidimensional analysis of pastes from 11 pear cultivars showed significant differences in color, nutritional composition, antioxidant activity, and free amino acids. PCA and PLS-DA indicated that genotype was the dominant source of variation, with PC1 explaining 84.1% of the total variance. This aligns with findings in strawberry [28] and citrus processing [29], where genetic background set baseline levels of phenolics, sugars, acids, and amino acids that were amplified during thermal processing. VIP analysis highlighted TPC, TFC, furfural, and M-TOTAL as key biomarkers for distinguishing cultivar quality, offering quantitative indicators for objective evaluation and traceability.

Raw material traits were decisive for sensory attributes. ‘Huangguan’ achieved the highest sensory score (83.33) and clarity (87.65%) and had the lowest soluble protein content (3.84 mg/kg). ‘Hwasan’ showed the lowest sensory score (59.67) and clarity (3.33%). These contrasts emphasize the roles of soluble protein, sugar–acid balance, and pectin state [30]. The exceptional clarity of ‘Huangguan’ is consistent with its low soluble solids (11.17%) and high juice yield (69.7%), suggesting depolymerized pectin and limited colloidal stability. Low-molecular-weight pectin with moderate acidity (1.27 g/L) promotes polyphenol–protein complexation and flocculation, facilitating co-removal of proteins and light-scattering particles during concentration [31]. This results in very low residual protein and minimal Maillard progression (browning degree, BD = 0.53) [6,7]. Protein-rich cultivars such as ‘Suli’ (37.58 mg/kg) retained higher turbidity and showed greater Maillard tendency. Elevated turbidity in ‘Hwasan’ (3.33%), ‘Yuluxiang’ (4.57%), and ‘Zhongli 1’ (5.00%) likely arises from high-molecular-weight, highly branched pectin forming stable colloidal networks that hinder sedimentation [30]. Suspended protein–polyphenol complexes may also contribute [32]. Cultivar differences in pectin structure, including degree of esterification and molecular weight distribution, further influence clarity [10], and the rheological properties of raw pectin can persist through concentration.

A cultivar–product matching strategy emerges from these quality profiles. ‘Hongzaosu’, with the highest TPC, TFC, and DPPH radical scavenging activity, suits antioxidant-oriented products aimed at mitigating oxidative stress. ‘Akizuki’ shows intense Maillard reactivity, fitting richly colored, caramel-like pastes that appeal to traditional flavor preferences. ‘Wanxiu’ offers a lustrous appearance, high amino acid content, and balanced overall quality, adding nutritional value. ‘Huangguan’ combines the highest transparency and sensory scores, making it optimal for premium, clarity-focused products with mild, pleasant taste. This genotype-driven alignment parallels differentiated development strategies in other fruit sectors [28].

Unlike the antioxidant-centric screening of Feng et al. [2], which favored high-phenolic cultivars, the present multidimensional evaluation integrated sensory attributes, processing suitability, and antioxidant properties. The lower-phenolic ‘Huangguan’ ranked highest in sensory acceptability, underscoring the primacy of clarity, golden appearance, and balanced flavor for consumer preference. Products from ‘Huangguan’ deliver a clean, bright appearance and agreeable taste, while ‘Hongzaosu’-based pastes offer enhanced antioxidant benefits. The use of vacuum concentration likely aided bioactive retention and browning control [12,13], highlighting the synergy between raw material selection and process optimization. Selecting cultivars according to product goals can meet diverse consumer needs, from health promotion to sensory appeal.

### 4.2. The Maillard Reaction Reshapes the Sensory and Antioxidant Profiles of Pear Paste

The Maillard reaction drives color, flavor, and antioxidant development during pear paste concentration [26,33]. Its intensity varies markedly among cultivars. For example, the high-sugar cultivar ‘Akizuki’ and high-amino acid cultivar ‘Wanxiu’ showed the greatest BD and MRPs. Their MRP profiles were rich in Strecker aldehydes and furan volatiles. Thus, substrate availability governs reaction intensity, with reducing sugars and certain amino acids being key [6,34]. Proline was detected only in ‘Akizuki’ (0.92 mg/g), which also had the highest BD (1.31). Proline is highly reactive and promotes melanoidin polymerization [35]. High sugar (770.2 g/kg) and lysine (0.52 mg/g) likely accelerated early Maillard stages [16,36]. In contrast, ‘Yuluxiang’ had the highest furfural (3831.21 µg/kg) and total MRPs (4113.44 µg/kg) but only moderate BD (0.91). This suggests a pathway favoring early intermediates over melanoidin formation [6]. Similar patterns occur in other fruit concentrates, where pentose availability and pH influence intermediate-polymer balance [37]. Our divergence likely reflects differences in pentose availability, free amino acids, pH, and water activity [37]. Reddish-brown hues in ‘Akizuki’ and ‘Hongzaosu’ are consistent with melanoidin formation. In contrast, ‘Huangguan’ had a bright golden appearance (BD = 0.53) due to its very low free amino acid pool (11.73 mg/g) and low soluble protein (3.84 mg/kg), which limit the reaction to early intermediates [37,38].

MRPs also reshape the antioxidant system. They include melanoidins, reductones, heterocyclic compounds (furans, pyrazines), and α-dicarbonyls. These act via radical scavenging (DPPH, ABTS, peroxyl), metal chelation (Fe^2+^, Cu^2+^), and lipid peroxidation inhibition [17,18,19]. Correlation analyses showed that ABTS and FRAP values were positively associated with TPC, TFC, and several MRPs, whereas DPPH was not. Hence, antioxidant capacity arises from a synergistic network of native polyphenols and processing-derived MRPs [19,20]. Similar synergy occurs in coffee and roasted barley extracts [39,40]. Assay discrepancies reflect different mechanisms. DPPH (organic media) is more sensitive to hydrophobic flavonoids [41]. ABTS and FRAP (aqueous media) better capture water-soluble activities from both phenolics and MRPs [39,40]. Therefore, ABTS and FRAP are recommended for comprehensive antioxidant assessment in pear paste, consistent with findings in apple juice [42].

For consumers, moderate browning (e.g., ‘Akizuki’, ‘Wanxiu’) provides rich caramel flavors and enhanced antioxidants, which help reduce oxidative stress and support wellness. However, the Maillard reaction is a double-edged sword: moderate heating boosts antioxidant activity [7,20], but excessive heating may form undesirable AGEs and acrylamide [35,40]. Thus, the balance between functional benefits and safety must be carefully managed. Optimized conditions (e.g., vacuum concentration) can maximize health benefits while minimizing risks.

### 4.3. Dual Roles of Amino Acid Profiles in Shaping Flavor and Functionality

‘Wanxiu’ and ‘Akizuki’ contained the highest total free amino acids (29.82 and 28.85 mg/g, respectively). They also had a diverse range of essential amino acids. This highlights their nutritional advantage. Several amino acids commonly found in pear paste (valine, leucine, isoleucine) correlated positively with ABTS activity. They also correlated with key Maillard-derived volatiles such as furfural and phenylacetaldehyde. These findings demonstrate a dual function during heating. First, these amino acids act as flavor precursors via Strecker degradation. For example, valine yields 2-methylpropanal. Leucine and isoleucine yield 3-methylbutanal and 2-methylbutanal. Methionine, detected in ‘Hwanggeum’, ‘Akizuki’, ‘Wanxiu’, and ‘Yuluxiang’, yields methional [34,43]. Second, sulfur- or imidazole-containing amino acids (e.g., methionine, histidine) can generate potent antioxidant products. Some amino acids also directly scavenge radicals [20,43]. Similar precursor roles have been reported in roasted coffee and baked cereals. In those products, amino acid composition dictated both aroma and antioxidant capacity [44].

Valine lacks known antioxidant functional groups. Therefore, its positive association with ABTS may reflect other roles. It could be a precursor of antioxidant heterocyclic compounds (e.g., pyrazines) [39]. It might also act synergistically with polyphenols. Alternatively, the association may arise from covariation with other factors. A model system coupled with LC-MS is needed to elucidate this mechanism. Specific amino acid-sugar combinations determine the structure and function of MRPs. For instance, lysine (0.18–0.52 mg/g across cultivars) can form the antioxidant compound furaneol [45]. Histidine (0.10–0.23 mg/g) can generate imidazopyridine compounds with fructose [20]. Proline polymerizes via pyrrolidine intermediates to form brown pigments [35]. Proline (0.92 mg/g) was found exclusively in ‘Akizuki’. This is consistent with its higher browning degree and ABTS activity. However, this cultivar-specific finding requires broader validation.

From a consumer perspective, amino acid-rich cultivars offer clear benefits. ‘Wanxiu’ and ‘Akizuki’ provide essential amino acids that support human nutrition. They also contribute to pleasant caramel-like aromas and enhanced antioxidant activity. The PLS-DA loadings (Figure 4c) distinguished two antioxidant chemotypes. Polyphenol-dominant cultivars (e.g., ‘Hongzaosu’, ‘Huangguan’) lay on the positive PC1 side. Flavor-associated cultivars (e.g., ‘Akizuki’, ‘Wanxiu’) lay on the negative side. The latter rely on amino acid-derived flavor compounds to achieve their sensory characteristics. This underscores how genotype governs processing pathways through precursor composition [28]. For product developers, selecting amino acid-rich cultivars allows the creation of flavorful, nutritious, and health-promoting pear pastes without adding external ingredients.

### 4.4. Limitations and Future Perspectives

This study systematically characterized pear pastes from 11 cultivars. It highlighted the decisive role of cultivar traits. Nevertheless, several limitations should be acknowledged. Only one standardized processing protocol was used. Therefore, the influence of key parameters remains unknown. These include concentration temperature, vacuum level, and pH adjustment. Their effects on cultivar traits need further study. Proline was detected only in ‘Akizuki’. Its association with browning was evident. However, this finding requires validation with a broader germplasm collection. Potentially harmful compounds (AGEs and acrylamide) were not quantified. This limits the benefit–risk assessment.

The sensory evaluation had two major limitations. The panel size was small (*n* = 9). The age range of the panelists was wide (25 to 50 years). With such a small sample, the wide age distribution reduces statistical power. It also limits the generalizability of the results. Future studies should recruit a larger panel. A more balanced age distribution would be beneficial. This would allow robust assessment of age-related preferences. It would also validate product acceptability across different consumer groups. Storage stability was not addressed. Consequently, temporal changes in color, flavor, antioxidant activity, and Maillard products remain unknown. The effects of storage conditions (temperature, light, packaging) on shelf life are also unknown.

Future research should focus on several key areas. Multi-omics approaches are needed to elucidate the molecular networks behind cultivar-specific quality differences. Systematic evaluation of processing variables would enable tailored processes for individual cultivars. It is also important to quantify safety-related markers and verify associated health benefits. Further investigation into storage stability is required to identify critical control points for different cultivars. Expanded germplasm screening will help establish a broader repository of specialized cultivars suitable for pear paste processing. Finally, targeted modeling systems, such as the amino acid–sugar Maillard reaction, will help analyze the specific contributions of individual amino acids. These include proline and valine, particularly their roles in browning and antioxidant activity.

## 5. Conclusions

This study establishes cultivar characteristics as the fundamental intrinsic factor governing pear paste quality. Multivariate analysis confirmed genotype as the primary determinant, with PC1 explaining 84.1% of the variance. Total phenolics, total flavonoids, furfural, and total Maillard products were identified as key biomarkers for cultivar differentiation. ‘Hongzaosu’ is ideal for high-antioxidant pastes due to its superior phenolic and flavonoid content and strong DPPH scavenging capacity. ‘Akizuki’ is suited for traditional pastes with deep color and rich flavor, owing to its pronounced Maillard reactivity and abundant Strecker aldehydes and furan-type volatiles. ‘Wanxiu’ offers balanced quality with a lustrous appearance and the highest free amino acid content. ‘Huangguan’ is optimal for premium sensory-focused products, achieving the highest clarity (87.65%) and sensory score (83.33). The Maillard reaction drives the formation of color, flavor, and antioxidant properties, and its products form a synergistic antioxidant network alongside native polyphenols and flavonoids. ABTS and FRAP are recommended as comprehensive methods for evaluating antioxidant capacity. The amino acid profile plays a dual role during thermal processing, acting as flavor precursors while also contributing to antioxidant activity. The cultivar–product precision matching strategy established here overcomes the limitations of traditional single-dimensional antioxidant-based evaluations and provides a scientific basis for the refined and differentiated development of the pear processing industry.

## Figures and Tables

**Table 1:** Fruit quality characteristics of pear cultivars.

Ripening Characteristics	Variety	Maturity Period	Firmness/(kg/cm^2^)	TSS/%	TA/(g/L)	Juice Yield/%
Early-season	Zhongli 1	Mid-to-late July	5.22 ± 0.13	11.53 ± 0.03	1.23 ± 0.02	69.49 ± 0.03
	Huangguan	Early-to-mid August	5.29 ± 0.04	11.17 ± 0.07	1.27 ± 0.00	69.7 ± 0.01
Mid-season	Wonhwang	Early-to-mid August	5.38 ± 0.07	12.50 ± 0.00	1.36 ± 0.03	71.73 ± 0.03
	Hwanggeum	Mid-August	5.80 ± 0.10	16.10 ± 0.00	1.24 ± 0.02	54.89 ± 0.01
	Hwasan	Late August	7.02 ± 0.19	13.27 ± 0.07	1.12 ± 0.00	67.95 ± 0.02
	Akizuki	Late August	5.24 ± 0.10	12.57 ± 0.03	1.77 ± 0.00	68.87 ± 0.01
	Yuluxiang	Late August	5.48 ± 0.13	12.67 ± 0.03	1.14 ± 0.00	70.54 ± 0.02
	Hongzaosu	Late August	5.58 ± 0.14	11.57 ± 0.03	2.45 ± 0.01	68.85 ± 0.01
Mid-to-late season	Hongxiangsu	Mid-September	7.65 ± 0.05	11.57 ± 0.03	0.85 ± 0.02	65.5 ± 0.03
	Suli	Mid-September	4.62 ± 0.10	11.07 ± 0.03	1.20 ± 0.01	60.71 ± 0.02
	Wanxiu	Early October	5.77 ± 0.09	9.57 ± 0.03	1.30 ± 0.01	58.72 ± 0.02

Note: TSS, total soluble solids; TA, titratable acidity. Values are presented as mean ± standard error (*n* = 3).

**Table 2:** Criteria for the sensory evaluation of pear paste.

Attribute (Max. Score)	Evaluation Criteria	Score Range
Taste (30)	Harmonious sweet-sour balance; full-bodied with a distinct pear flavor; smooth texture with a pleasant aftertaste.	21~30
	One dimension (sweet/sour) dominates; simplistic flavor; slightly gritty or sticky mouthfeel.	11~20
	Cloyingly sweet or sharply acidic; off-flavors (burnt, bitter, astringent); gritty, hard to swallow.	0~10
Aroma (30)	Intense, pure, and pleasant aroma of stewed/preserved pear; absent of off-odors.	21~30
	Faint pear aroma; slight caramelized or sugary notes; no unpleasant smells.	11~20
	Lack of pear fragrance; distinct off-odors (smoky, fermented, stale).	0~10
Color (20)	Natural (amber/golden-brown), luminous, and translucent.	15~20
	Too light or dark; moderately glossy; acceptable.	8~14
	Dull, dark, or uneven; opaque.	0~7
Texture (20)	Homogeneous, viscous paste; ideal flowability; free of impurities, crystals, or separation.	15~20
	Slightly too thin or thick; minor sedimentation/crystals, reversible by stirring.	8~14
	Inhomogeneous; prominent crystals, severe separation, or foreign material.	0~7

**Table 3:** Color parameters and appearance of pear paste from different cultivars.

Serial No.	Cultivar	*L* ^*^	*a* ^*^	*b* ^*^	*C* ^*^	*h*°	Clarity/%
1	Hwanggeum	26.64 ± 0.01 ^d^	1.93 ± 0.03 ^c^	9.95 ± 0.001 ^f^	10.13 ± 0.01 ^e^	78.99 ± 0.19 ^g^	7.50 ± 0.19 ^f^
2	Wonhwang	26.56 ± 0.08 ^d^	2.55 ± 0.11 ^b^	10.34 ± 0.14 ^e^	10.65 ± 0.16 ^d^	76.16 ± 0.37 ^h^	14.90 ± 1.6 ^e^
3	Hwasan	29.34 ± 0.03 ^a^	0.59 ± 0.04 ^d^	12.59 ± 0.06 ^b^	12.61 ± 0.06 ^b^	87.34 ± 0.19 ^f^	3.33 ± 0.05 ^g^
4	Akizuki	24.20 ± 0.05 ^f^	4.11 ± 0.08 ^a^	10.08 ± 0.14 ^ef^	10.89 ± 0.15 ^d^	67.83 ± 0.25 ^i^	22.45 ± 0.21 ^d^
5	Wanxiu	28.72 ± 0.07 ^b^	0.26 ± 0.02 ^e^	13.17 ± 0.10 ^a^	12.97 ± 0.10 ^a^	88.87 ± 0.08 ^e^	51.33 ± 0.27 ^b^
6	Hongzaosu	26.10 ± 0.03 ^e^	4.03 ± 0.12 ^a^	9.10 ± 0.07 ^g^	9.95 ± 0.12 ^e^	66.15 ± 0.47 ^j^	8.14 ± 0.38 ^f^
7	Suli	28.63 ± 0.07 ^b^	−1.02 ± 0.02 ^g^	12.04 ± 0.10 ^c^	12.09 ± 0.01 ^c^	94.84 ± 0.13 ^b^	22.40 ± 0.28 ^d^
8	Zhongli 1	28.23 ± 0.15 ^c^	−0.38 ± 0.07 ^f^	10.94 ± 0.07 ^d^	10.95 ± 0.08 ^d^	91.97 ± 0.36 ^c^	5.00 ± 0.18 ^g^
9	Yuluxiang	26.57 ± 0.04 ^d^	1.90 ± 0.14 ^c^	10.75 ± 0.07 ^d^	10.92 ± 0.09 ^d^	80.00 ± 0.70 ^g^	4.57 ± 0.05 ^g^
10	Hongxiangsu	28.63 ± 0.14 ^b^	−0.11 ± 0.05 ^f^	12.08 ± 0.08 ^c^	12.08 ± 0.08 ^c^	90.52 ± 0.25 ^d^	28.43 ± 0.09 ^c^
11	Huangguan	29.09 ± 0.17 ^a^	−1.65 ± 0.04 ^h^	8.19 ± 0.03 ^h^	8.36 ± 0.03 ^f^	101.38 ± 0.32 ^a^	87.65 ± 0.12 ^a^

Note: L^*^, a^*^, b^*^, C^*^, and *h*° are dimensionless color parameters. Values are presented as mean ± standard error (*n* = 3). Different lowercase letters within the same column indicate significant differences (*p* < 0.05). The same applies below.

**Table 4:** Nutritional composition and sensory quality of pear paste from different pear cultivars.

Cultivar	SS/(g/kg)	TA/%	Vc/(mg/kg)	SP/(mg/kg)	TFC/(mg/kg)	TPC/(mg/kg)	Sensory Evaluation (Score)
Hwanggeum	668.8 ± 22.9 ^abcd^	1.18 ± 0.01 ^fg^	37.65 ± 0.88 ^a^	31.99 ± 0.31 ^bc^	657.4 ± 15.6 ^cd^	1346.4 ± 7.0 ^c^	71.33 ± 5.90 ^ab^
Wonhwang	742.5 ± 18.1 ^ab^	1.40 ± 0.01 ^d^	19.58 ± 0.79 ^d^	23.25 ± 0.38 ^d^	571.9 ± 6.2 ^de^	1215.4 ± 19.8 ^cd^	65.33 ± 2.73 ^b^
Hwasan	633.7 ± 7.0 ^bcd^	1.10 ± 0.01 ^g^	14.74 ± 0.16 ^ef^	23.16 ± 0.33 ^d^	478.1 ± 10.7 ^e^	1052.8 ± 38.5 ^d^	59.67 ± 2.60 ^b^
Akizuki	770.2 ± 35.6 ^a^	1.49 ± 0.01 ^c^	28.81 ± 0.21 ^b^	28.62 ± 0.30 ^c^	869.8 ± 8.9 ^b^	1738.9 ± 15.0 ^b^	65.67 ± 3.76 ^b^
Wanxiu	735.1 ± 44.4 ^ab^	1.70 ± 0.01 ^b^	23.77 ± 0.57 ^c^	34.02 ± 0.76 ^b^	841.7 ± 23.4 ^b^	1886.0 ± 67.0 ^ab^	73.00 ± 2.65 ^ab^
Hongzaosu	673.6 ± 15.3 ^abcd^	1.95 ± 0.00 ^a^	15.81 ± 0.26 ^e^	21.18 ± 0.59 ^d^	1747.9 ± 51.5 ^a^	1997.6 ± 73.7 ^a^	72.00 ± 1.15 ^ab^
Suli	695.4 ± 11.3 ^abc^	0.89 ± 0.02 ^i^	24.17 ± 0.68 ^c^	37.58 ± 1.49 ^a^	910.2 ± 28.3 ^b^	1777.4 ± 48.9 ^b^	72.67 ± 1.33 ^ab^
Zhongli 1	653.6 ± 20.3 ^bcd^	1.23 ± 0.01 ^ef^	13.17 ± 0.23 ^f^	31.40 ± 1.24 ^bc^	585.7 ± 16.5 ^de^	1180.0 ± 10.6 ^cd^	71.67 ± 1.86 ^ab^
Yuluxiang	510.8 ± 30.9 ^e^	1.27 ± 0.02 ^e^	20.46 ± 0.83 ^d^	23.48 ± 0.10 ^d^	766.9 ± 69.3 ^bc^	1704.0 ± 41.9 ^b^	61.33 ± 2.19 ^b^
Hongxiangsu	574.0 ± 8.8 ^de^	0.98 ± 0.01 ^h^	15.91 ± 0.72 ^e^	31.08 ± 0.64 ^bc^	640.5 ± 22.4 ^cd^	1356.6 ± 18.2 ^c^	69.67 ± 1.86 ^ab^
Huangguan	596.9 ± 7.6 ^cde^	1.12 ± 0.03 ^fg^	12.74 ± 0.96 ^f^	3.84 ± 0.39 ^e^	863.6 ± 11.3 ^b^	1246.2 ± 34.0 ^cd^	83.33 ± 0.88 ^a^

Note: SS, soluble sugars; TA, titratable acidity; Vc, vitamin C; SP, soluble protein; TFC, total flavonoid content; TPC, total phenolic content. Sensory scores are on a 0–100 scale. Values are presented as mean ± standard error (*n* = 3). Different lowercase letters in the same column indicate significant differences (*p* < 0.05) as determined by means of one-way ANOVA followed by Tukey’s HSD post hoc test.

**Table 5:** Composition Maillard Reaction Products of different pear paste cultivars µg/kg.

Order	Compound	Hwanggeum	Wonhwang	Hwasan	Akizuki	Wanxiu	Hongzaosu	Suli	Zhongli 1	Yuluxiang	Hongxiangsu	Huangguan
1	Acetaldehyde	5.39 ± 0.99	1.01 ± 0.01	2.48 ± 0.97	1.17 ± 0.00	0.88 ± 0.11	1.90 ± 0.88	1.90 ± 0.50	1.27 ± 0.26	-	2.01 ± 0.15	1.41 ± 0.39
2	Propanal, 2-methyl-	4.70 ± 0.65	-	-	19.52 ± 5.22	19.35 ± 1.23	20.84 ± 6.35	16.37 ± 3.99	-	-	-	4.99 ± 0.33
3	Butanal,3-methyl-	-	-	-	42.29 ± 3.97	-	11.20 ± 1.68	-	-	5.70 ± 0.00	-	-
4	Pentanal	-	-	6.34 ± 0.09	-	-	-	7.31 ± 2.13	-	-	-	-
5	Butanal, 2-methyl-	-	-	-	-	22.05 ± 6.68	-	-	-	-	-	-
6	Furfural	805.32 ± 38.62	406.65 ± 25.33	1457.99 ± 106.11	1572.51 ± 180.55	255.85 ± 1.37	421.32 ± 48.88	901.31 ± 22.62	397.71 ± 11.60	3831.21 ± 371.26	1231.81 ± 72.04	2223.90 ± 158.96
7	Methional	-	3.44 ± 0.05	-	56.76 ± 7.89	3.76 ± 0.03	15.54 ± 2.21	5.35 ± 0.32	7.35 ± 0.01	-	3.64 ± 0.22	-
8	2-Furancarboxaldehyde, 5-methyl-	199.31 ± 9.78	30.52 ± 1.09	105.42 ± 19.18	76.79 ± 6.39	60.56 ± 5.46	14.89 ± 1.59	42.61 ± 5.46	29.61 ± 0.11	93.77 ± 6.23	27.78 ± 5.40	28.68 ± 3.48
9	Benzeneacetaldehyde	-	-	-	8.60 ± 2.41	-	-	-	-	12.90 ± 1.15	-	18.47 ± 4.64
10	2-Furanmethanol	21.02 ± 5.78	11.20 ± 0.23	11.82 ± 4.40	7.63 ± 0.59	8.01 ± 0.51			5.22 ± 2.56	25.36 ± 14.32	12.97 ± 2.78	
11	Ethanone, 1-(2-furanyl)-	25.72 ± 2.36	-	10.74 ± 2.13	-	-	-	-	-	124.67 ± 19.40	3.02 ± 0.13	26.29 ± 4.15
12	Pyrazine, 2,6-dimethyl-	-	-	-	7.71 ± 0.77	5.77 ± 0.42	-	-	-	-	-	-
13	Furan, 2-pentyl-	23.37 ± 1.54	-	-	-	-	-	-	-	19.83 ± 2.55	-	-
14	1-Propanone, 1-(2-furanyl)-	-	10.63 ± 0.12	92.07 ± 5.69	-	-	107.95 ± 11.01	-	-	-	3.36 ± 0.05	-
15	Ethanone, 1-(1H-pyrrol-2-yl)-	30.35 ± 2.54	-	24.31 ± 1.24	-	8.38 ± 1.11	4.04 ± 0.22	20.42 ± 2.85	-	-	-	-
16	M-TOTAL	1115.18	463.45	1711.17	1792.98	384.61	597.68	995.27	439.89	4113.44	1284.59	2303.74
17	BD (OD_420_)	0.87 ± 0.01 ^c^	0.89 ± 0.01 ^c^	0.74 ± 0.02 ^e^	1.31 ± 0.01 ^a^	0.72 ± 0.01 ^e^	1.01 ± 0.01 ^b^	0.79 ± 0.01 ^d^	0.79 ± 0.02 ^d^	0.91 ± 0.02 ^c^	0.88 ± 0.01 ^c^	0.53 ± 0.02 ^f^

Note: Values are presented as mean ± standard error (*n* = 3). For the browning degree (BD), statistical analysis was performed using one-way ANOVA followed by Tukey’s HSD post hoc test; different lowercase letters in the same row indicate significant differences (*p* < 0.05). For the BD, statistical BD is expressed as absorbance at 420 nm (OD_420_, dimensionless). “-” indicates not detected.

**Table 6:** Composition Free Amino acid of different pear paste cultivars mg/g.

Free Amino Acids	Hwanggeum	Wonhwang	Hwasan	Akizuki	Wanxiu	Hongzaosu	Suli	Zhongli 1	Yuluxiang	Hongxiangsu	Huangguan
Thr *	-	0.08 ± 0.00 ^b^	-	0.19 ± 0.01 ^a^	-	0.07 ± 0.01 ^b^	-	0.08 ± 0.00 ^b^	0.19 ± 0.03 ^a^	0.14 ± 0.03 ^ab^	-
Val *	1.03 ± 0.04 ^bc^	0.96 ± 0.02 ^bcd^	1.17 ± 0.10 ^b^	1.50 ± 0.04 ^a^	1.19 ± 0.06 ^b^	0.85 ± 0.08 ^cd^	0.83 ± 0.03 ^cd^	0.76 ± 0.04 ^d^	0.88 ± 0.05 ^cd^	0.86 ± 0.01 ^cd^	0.95 ± 0.02 ^bcd^
Met *	0.09 ± 0.01 ^a^	-	-	0.12 ± 0.01 ^a^	0.12 ± 0.01 ^a^	-	-	-	0.12 ± 0.01 ^a^	-	-
Ile *	0.39 ± 0.01 ^b^	0.41 ± 0.02 ^b^	0.25 ± 0.02 ^d^	0.68 ± 0.01 ^a^	0.61 ± 0.02 ^a^	0.23 ± 0.04 ^d^	0.28 ± 0.00 ^cd^	0.37 ± 0.01 ^bc^	0.39 ± 0.01 ^b^	0.23 ± 0.03 ^d^	0.37 ± 0.02 ^bc^
Leu *	0.31 ± 0.01 ^d^	0.10 ± 0.00 ^g^	0.16 ± 0.00 ^efg^	0.89 ± 0.01 ^a^	0.65 ± 0.03 ^b^	0.14 ± 0.02 ^fg^	0.37 ± 0.01 ^cd^	0.30 ± 0.04 ^de^	0.51 ± 0.01 ^bc^	0.24 ± 0.04 ^defg^	0.27 ± 0.07 ^def^
Phe *	0.35 ± 0.03 ^bcd^	0.15 ± 0.03 ^d^	0.46 ± 0.06 ^abc^	0.62 ± 0.01 ^a^	0.61 ± 0.08 ^a^	0.33 ± 0.07 ^cd^	0.31 ± 0.01 ^cd^	0.37 ± 0.03 ^bc^	0.55 ± 0.02 ^ab^	0.32 ± 0.03 ^cd^	0.38 ± 0.04 ^bc^
Lys *	0.22 ± 0.01 ^ef^	0.18 ± 0.03 ^f^	0.20 ± 0.03 ^ef^	0.52 ± 0.00 ^a^	0.47 ± 0.01 ^ab^	0.18 ± 0.01 ^f^	0.29 ± 0.02 ^de^	0.33 ± 0.02 ^d^	0.42 ± 0.01 ^bc^	0.32 ± 0.02 ^d^	0.34 ± 0.04 ^cd^
Asp	7.82 ± 0.12 ^f^	12.66 ± 0.03 ^c^	11.96 ± 0.19 ^c^	17.47 ± 0.04 ^b^	22.38 ± 0.07 ^a^	10.14 ± 0.43 ^d^	10.85 ± 0.05 ^d^	9.01 ± 0.08 ^e^	5.80 ± 0.13 ^g^	8.76 ± 0.06 ^e^	6.25 ± 0.00 ^g^
Ser	0.92 ± 0.02 ^a^	0.35 ± 0.01 ^d^	0.30 ± 0.00 ^de^	0.45 ± 0.01 ^c^	0.70 ± 0.00 ^b^	0.21 ± 0.02 ^f^	0.26 ± 0.01 ^ef^	0.27 ± 0.00 ^ef^	0.52 ± 0.03 ^c^	0.30 ± 0.02 ^de^	0.76 ± 0.01 ^b^
Glu	0.95 ± 0.03 ^f^	1.12 ± 0.03 ^de^	0.62 ± 0.02 ^g^	1.63 ± 0.02 ^a^	1.43 ± 0.01 ^b^	1.00 ± 0.02 ^f^	1.24 ± 0.02 ^c^	1.06 ± 0.00 ^ef^	0.99 ± 0.01 ^f^	0.61 ± 0.04 ^g^	1.20 ± 0.02 ^cd^
Gly	0.22 ± 0.01 ^de^	0.21 ± 0.01 ^e^	0.22 ± 0.01 ^de^	0.51 ± 0.01 ^b^	0.58 ± 0.01 ^a^	0.19 ± 0.01 ^e^	0.36 ± 0.01 ^c^	0.26 ± 0.01 ^d^	0.32 ± 0.01 ^c^	0.26 ± 0.01 ^d^	0.32 ± 0.01 ^c^
Ala	0.22 ± 0.00 ^ef^	0.36 ± 0.01 ^c^	0.16 ± 0.03 ^fg^	0.90 ± 0.01 ^a^	0.60 ± 0.03 ^b^	0.11 ± 0.02 ^g^	0.31 ± 0.01 ^cde^	0.26 ± 0.01 ^de^	0.28 ± 0.01 ^cde^	0.29 ± 0.01 ^cde^	0.35 ± 0.02 ^cd^
Tyr	-	0.18 ± 0.06 ^cd^	0.23 ± 0.05 ^bcd^	0.46 ± 0.03 ^a^	0.13 ± 0.01 ^cd^	0.11 ± 0.01 ^d^	0.37 ± 0.04 ^ab^	0.12 ± 0.01 ^d^	0.11 ± 0.01 ^d^	0.29 ± 0.04 ^bc^	0.22 ± 0.01 ^bcd^
His	0.10 ± 0.00 ^d^	0.11 ± 0.01 ^cd^	0.12 ± 0.01 ^bcd^	0.23 ± 0.01 ^a^	0.22 ± 0.02 ^a^	0.14 ± 0.01 ^bcd^	-	0.13 ± 0.00 ^bcd^	0.18 ± 0.01 ^ab^	0.14 ± 0.01 ^bcd^	0.16 ± 0.01 ^bc^
Arg	-	-	0.11 ± 0.01 ^c^	0.27 ± 0.00 ^a^	0.13 ± 0.01 ^c^	-	-	0.13 ± 0.02 ^cd^	0.25 ± 0.02 ^ab^	0.18 ± 0.03 ^abc^	0.16 ± 0.04 ^abc^
Pro	-	-	-	0.92 ± 0.06	-	-	-	-	-	-	-
TAA	12.62 ± 0.21 ^ef^	16.87 ± 0.14 ^c^	15.96 ± 0.24 ^cd^	28.85 ± 1.49 ^b^	29.82 ± 0.78 ^a^	13.89 ± 0.39 ^e^	15.46 ± 0.12 ^d^	13.44 ± 0.14 ^e^	11.52 ± 0.26 ^f^	12.95 ± 0.18 ^e^	11.73 ± 0.10 ^f^
EAA	2.39 ± 0.05 ^d^	1.80 ± 0.03 ^f^	2.24 ± 0.06 ^de^	4.33 ± 0.05 ^a^	3.65 ± 0.02 ^b^	1.74 ± 0.09 ^f^	2.08 ± 0.04 ^def^	2.13 ± 0.07 ^de^	2.88 ± 0.06 ^c^	1.97 ± 0.05 ^ef^	2.32 ± 0.06 ^de^
NEAA	10.23 ± 0.16 ^gh^	14.99 ± 0.15 ^c^	13.72 ± 0.18 ^d^	22.83 ± 0.16 ^b^	26.17 ± 0.04 ^a^	11.89 ± 0.47 ^e^	13.38 ± 0.09 ^d^	11.23 ± 0.09 ^ef^	8.44 ± 0.19 ^i^	10.75 ± 0.05 ^fg^	9.41 ± 0.05 ^h^

Note: Values are presented as mean ± standard error (*n* = 3). Statistical analysis was performed using one-way ANOVA followed by Tukey’s HSD post hoc test; different lowercase letters in the same row indicate significant differences (*p* < 0.05). Asterisk (*) indicates essential free amino acids. Dash (-) indicates that the free amino acid was not detected or was below the detection limit. Abbreviations: TAA, total free amino acids; EAA, essential free amino acids; NEAA, non-essential free amino acids.

## Data Availability

The original contributions presented in this study are included in the article. Further inquiries can be directed to the corresponding author.
